# Association of Nutritional Support With Clinical Outcomes in Malnourished Cancer Patients: A Population-Based Matched Cohort Study

**DOI:** 10.3389/fnut.2020.603370

**Published:** 2021-03-10

**Authors:** Nina Kaegi-Braun, Philipp Schuetz, Beat Mueller, Alexander Kutz

**Affiliations:** ^1^Division of Endocrinology, Diabetes and Metabolism, University Department of Medicine, Kantonsspital Aarau, Aarau, Switzerland; ^2^Division of General and Emergency Medicine, University Department of Medicine, Kantonsspital Aarau, Aarau, Switzerland; ^3^Faculty of Medicine, University of Basel, Basel, Switzerland

**Keywords:** malnutrition, nutritional support, cancer, oncology, mortality

## Abstract

Malnutrition is prevalent in hospitalized cancer patients and has been associated with poor therapy response and unfavorable clinical outcome. While recent studies have shown a survival benefit through nutritional support in a hospitalized malnourished medical population including cancer patients, we aimed to investigate the association of nutritional support with in-hospital mortality and other clinical outcomes in a nationwide inpatient cancer population. In this population-based cohort study, using a large Swiss administrative claims database from April 2013 to December 2018, we created two cohorts of malnourished cancer patients on medical wards. We generated two pairwise cohorts of malnourished patients who received nutritional support by 1:1 propensity-score matching to patients not receiving nutritional support. The primary outcome was all-cause in-hospital mortality. Secondary outcomes were 30-days all-cause hospital readmission and discharge to a post-acute care facility. To account for disease activity, we stratified patients either admitted for cancer as main diagnosis or admitted with cancer as comorbidity. Among 1,851,498 hospitalizations on medical ward, we identified a total of 32,038 malnourished cancer patients. After matching, 11,906 (37%) cases were included in the “cancer main diagnosis cohort” and 5,954 (18.6%) in the “cancer comorbidity cohort.” Patients prescribed a nutritional support showed a lower in-hospital mortality in both cohorts as compared to their respective matched controls not receiving nutritional support [cancer main diagnosis cohort: 15.4 vs. 19.4 %, OR 0.76 (95% CI 0.69–0.83); cancer comorbidity cohort: 7.4 vs. 10.2%, OR 0.71 (95% CI 0.59–0.85)]. While we found no difference in 30-days readmission rates, discharge to a post-acute care facility was less frequent in the nutritional support group of both cohorts. In this large cohort study, nutritional support in hospitalized patients with either cancer as main diagnosis or comorbidity was associated with a lower risk of in-hospital mortality and discharge to a post-acute care facility.

## Introduction

In cancer patients, the combination of pathophysiological changes in appetite signals, treatment side effects, as well as physical limitations results in reduced food intake and consequently puts these patients at high risk for malnutrition (1). This state of chronic disease-related malnutrition is called cachexia (2). Cachexia is not only caused by anorexia but also by a state of metabolic derangements due to catabolic drivers such as inflammatory cytokines, which finally lead to a state of weight loss and sarcopenia.

Malnutrition and its consequences is more frequent in the population of patients with malignant diseases. Prevalences range from 20 to 70% in worldwide studies, depending on age, entity of the cancer, type of treatment and cancer stages (1). For example, a French study found an overall prevalence of 39% in cancer patients. There were even higher prevalence rates in patients with head neck cancer (48.9%), esophageal/stomac cancer (60.2%), and pancreatic cancer (66.7%) (3). In a recently published randomized controlled trial investigating the effect of nutritional support in medical inpatinets (4), cancer was the second most frequent admission diagnosis in patients at nutritional risk (18.5%).

The impact of malnutrition in cancer patients on health and financial factors had been shown in several studies: Malnourished cancer patients showed increased mortality rates (5–7), as well as longer length of hospital stay (5, 7, 8), higher health care cost (8), lower scores in QoL (9), and lower tolerance to chemotherapy (6).

Because of the strong impact of malnutrition on prognosis in cancer patients, the current ESPEN evidence-based nutrition guidelines (10) highlight the importance of recognizing and assessing malnutrition followed by treating it adequately with an individualized nutritional treatment concept. However, evidence for nutritional treatment to improve clinical outcomes in cancer patients is heterogenous. In 2012, a meta-analysis was showing that oral nutritional interventions increase the nutritional intake and improve some aspects of quality of life, but do not affect mortality risk (11). Focusing on patients with chemo(radio)therapy, another review revealed benefit of nutritional interventions on body weight but not regarding treatment toxicity or survival (12). A randomized controlled trial investigating long-term outcomes in patients with colorectal cancer showed a beneficial effect of individualized nutritional support counseling on nutritional status, survival as well as treatment toxicity (13).

To substantiate evidence in cancer patients, the ESPEN guidelines also recommend the performance of further trials to investigate the efficacy of nutritional interventions (10).

In Switzerland, screening, assessment, and treatment of malnutrition has remarkably increased during the recent years not only due to improved awareness of malnutrition being a predictor of adverse outcome but also due to reimbursement reasons^1^.

The aim of this study was to investigate the association of nutritional support with in-hospital mortality and other clinical outcomes in malnourished cancer patients as treated in clinical routine using “real-world” data.

## Materials and Methods

### Study Design

This was a retrospective cohort study using a nation-wide population-based database from Switzerland to investigate the association between nutritional support and clinical outcomes in malnourished cancer patients. We used administrative claims data from the Federal Statistical Office of Switzerland (“Medizinstatistik”)^1^ and observation period was from April 2013 to December 2018. The database contains all Swiss inpatient discharge records from acute care-, general-, and specialty hospitals, and provides information about in-hospital health-care use, main diagnosis and comorbidities, diagnostic tests and procedures. Data assessment was longitudinal with each patient having a unique code, so that re-hospitalization could be tracked and one patient could have more than one index hospitalization. Main diagnoses and comorbidities were assessed during index hospitalization and coded using the International Classification of Disease, version 10, German Modification (ICD-10-GM) codes; in-hospital therapeutic procedures were recorded using the “Swiss classification of operation” codes (CHOP).

The institutional review board of Northwestern Switzerland (AG/SO 2009/074 and EKNZ BASEC PB_2017-00449) approved the trial, including waiver of informed consent of the patients because data was de-identified. We followed the Strengthening the Reporting of Observational Studies in Epidemiology (STROBE) reporting guidelines (14).

### Participants

We included adult hospitalizations (≥18 years) on medical wards who were malnourished and had a diagnosis of cancer. Patients with an admission to intensive care unit in the course of hospitalization were excluded to increase specify of the study population. Malnutrition was defined as the presence of one of the following ICD-10-GM codes: E43 for unspecified severe protein-energy malnutrition, E44 for protein-energy malnutrition of moderate and mild degree, and E46 for unspecified protein-energy malnutrition^2^. Appendix Table 1 shows detailed information about the ICD-10 codes of malnutrition. For the assessment of cancer diagnosis and specific type of cancer we also used ICD-10-GM codes (C00-97). We stratified our cohort in two groups depending on whether the reason for admission cancer (“cancer main diagnosis cohort”) or whether cancer was coded as a comorbidity (“cancer comorbidity cohort”). Using the ICD-10-GM, we also sub classified the patients according to type of tumor: gastrointestinal (C15–C26), respiratory (C30–C39), hematological (C81–C96), urogenital (C51–C66), breast (C50), and others (all other ICD-10-GM codes for cancer; i.e., oropharyngeal, bone, skin, mesothelial, cerebral, endocrinological and unknown origin). Regarding current treatment of cancer we categorized the patients by means of CHOP codes: chemotherapy (99.25.xx), immunotherapy (99.28.xx), stem cell transplantation (41.0x.xx), radiotherapy (92.2x.xx, 92.3x.xx, 92.4x.xx).

### Exposure

We screened eligible hospitalizations of malnourished cancer patients for the presence of nutritional support during hospitalization. Again, we used CHOP codes to classify patients with nutritional support (“89.0A.32” and “89.0A.4x” for dietary advice and/or nutritional therapy, “96.6” for enteral tube-feeding of concentrated nutrients, and “99.15” for parenteral infusion of concentrated nutrient solutions). Dietary advice and nutritional therapy were not further classified; general practice in Switzerland is based on assessment and advice of a dietician and adjustment of hospital meals, food fortification, additional snacks or the use of oral nutritional supplements (ONS).

### Outcomes

Our primary outcome was all-cause in-hospital mortality. Secondary outcomes were 30-days readmission rate from any cause as well as discharge to a post-acute care facility, defined as every discharge location except from home.

### Statistical Analysis

Before and after propensity-score matching we used descriptive statistics and standardized difference were applied to compare baseline characteristics of patients stratified by nutritional support. A standardized difference of <10% indicated adequate balance between groups (15).

We fitted different logistic regression models to control for confounding and to assess the robustness of our results. In a first step, we performed a unadjusted logistic regression analysis for primary and secondary outcomes comparing hospitalizations of patients on nutritional support with those not on nutritional support. Second, we fitted a multivariable logistic regression model adjusting for sociodemographic factors (age, gender, nationality, insurance status, month and year of admission, location before and mode of admission, hospital size, hospital site). Third, in the fully-adjusted model we included the sociodemographic factors as well as main diagnoses, comorbidities, entity of cancer, type of cancer therapy, severity of malnutrition, the total amount of hospitalizations per patient, any palliative treatment, Charlson Comorbidity Index, Hospital Frailty Risk Score, and length of hospital stay. Finally, we performed a propensity-score matched model by matching hospitalizations of patients receiving nutritional support 1:1 to a cohort of hospitalizations without nutritional support. Doing so, we used a multivariable logistic regression model to calculate the probability of receiving nutritional support and as covariates we used all parameters from the fully adjusted model (except for hospital site). The estimated propensity-score was used for the matching with nearest neighbor controls and the caliper size was 0.0005. Discrimination analysis revealed a c-index of 0.7545 for the final model. After propensity-score matching we performed logistic regressions, which were adjusted for hospital site.

For the secondary analyses and assessment of 30-days readmission and discharge to post-acute care facility, we excluded hospitalizations with an event of in-hospital death to address competing risk. Kaplan-Meier curves were used to illustrate the differences in time to in-hospital mortality and cox-regression model were used to calculate the hazard ratio.

In a sensitivity analysis, we explored the association between nutritional support and outcomes of interest in patients with cancer as main admission diagnosis only. We performed subgroup analyses to explore differences among cancer entities and treatments of cancer by calculating effect modification.

We used STATA, version 15.1 (StataCorp LLC), for all analyses. *P*-values are two-sided and values <0.05 are considered as significant.

## Results

**Figure 1:**
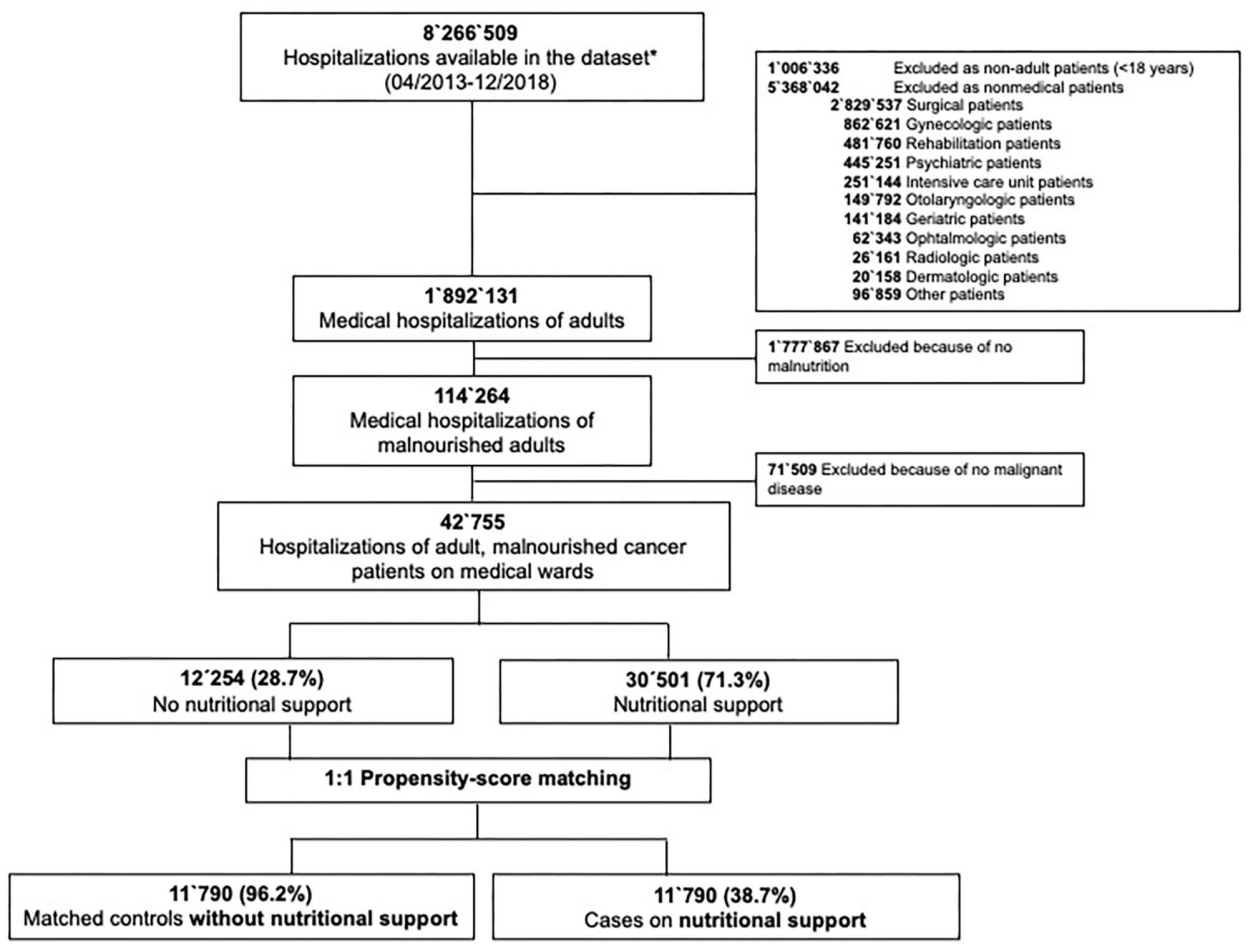
Study flow sheet.

In total, our database included 8,266,509 hospitalizations from April 2013 to December 2018 (Figure 1). Among them, we identified 1,892,131 hospitalizations of adult patients on medical wards. 6% (114,264) of them had a code for malnutrition and among the malnourished cohort 37.4% (42,755) had a malignancy. Nutritional support was provided in 71.3% (*n* = 30,501) of these cases, whereas 28.7% (*n* = 12,254) had no code for nutritional support. Even in the unmatched population most baseline characteristics were quite well-balanced. Importantly, there was a difference in the incidence of severe malnutrition (36.5% in the nutritional support group vs. 13.2% in the control group) and total length of hospital stay (median LOS 4 days longer in the nutritional support group).

After 1:1 propensity score matching, we paired 38.7% of the cases with nutritional support to 96.2% of the hospitalizations without nutritional support. All baseline characteristics were well-balanced (Table 1). Mean age was 70.3 years, 43.7% of the patients were female and most patients were treated in tertiary care hospitals (77.7%). 68.2% of the patients were admitted because of their malignancy. Burden of comorbidity was high as reflected by a mean Charlson Comorbidity index of 6.8 points (*SD* ± 3.1 points). 13.9% of the overall matched population were severely malnourished.

**Table 1:** Baseline characteristics before and after 1:1 propensity-score matching stratified by nutritional support.

**Overall Population With Cancer**	**Before Matching**			**After Matching**		
	**No nutritional Support**	**Nutritional Support**	**Std (%)**	**No Nutritional Support**	**Nutritional Support**	**Std (%)**
*n*	12,254	30,501		11,790	11,790	
**Sociodemographics**						
Age, mean (*SD*)	70.4 (12.9)	69.5 (12.7)	6.82	70.3 (12.9)	70.2 (12.2)	0.34
Female (%)	5,360 (43.7)	13,611 (44.6)	−1.78	5,171 (43.9)	5,121 (43.4)	0.86
Swiss resident (%)	10,199 (83.2)	26,049 (85.4)	5.98	9,843 (83.5)	9,870 (83.7)	0.62
Public insurance (%)	9,287 (75.8)	23,281 (76.3)	1.27	8,938 (75.8)	9,012 (76.4)	1.47
Emergency admission (%)	7,824 (63.8)	20,557 (67.4)	−7.48	7,624 (64.7)	7,590 (64.4)	0.60
Admission from home (%)	10,287 (83.9)	26,688 (87.5)	−10.16	10,003 (84.8)	9,970 (84.6)	0.78
Tertiary hospital (%)	9,387 (76.6)	25,575 (83.8)	−18.27	9,223 (78.2)	9,095 (77.1)	2.61
**Main Diagnosis (%)**						
Endocrinology	204 (1.7)	675 (2.2)	−3.98	202 (1.7)	211 (1.8)	−0.58
Cardiology	467 (3.8)	955 (3.1)	3.72	439 (3.7)	456 (3.9)	−0.76
Infectiology	371 (3.0)	841 (2.8)	1.61	361 (3.1)	361 (3.1)	0.00
Pneumology	802 (6.5)	1,669 (5.5)	4.52	758 (6.4)	782 (6.6)	−0.82
Oncology	8,336 (68.0)	21,525 (70.6)	−5.52	8,047 (68.3)	8,027 (68.1)	0.36
Psychiatry	116 (0.9)	144 (0.5)	5.66	91 (0.8)	89 (0.8)	0.20
Neurology	82 (0.7)	164 (0.5)	1.70	73 (0.6)	74 (0.6)	−0.11
Gastroenterology	617 (5.0)	1,681 (5.5)	−2.13	601 (5.1)	595 (5.0)	0.23
Musculoskeletal	118 (1.0)	296 (1.0)	−0.08	112 (0.9)	116 (1.0)	−0.35
Nephrology	249 (2.0)	540 (1.8)	1.92	244 (2.1)	236 (2.0)	0.48
**Comorbidities (%)**						
Diabetes	1,934 (15.8)	4,789 (15.7)	0.22	1,867 (15.8)	1,896 (16.1)	−0.67
Coronary heart disease	1,343 (11.0)	3,553 (11.6)	−2.18	1,308 (11.1)	1,300 (11.0)	0.22
Hypertension	4,173 (34.1)	10,973 (36.0)	−4.03	4,077 (34.6)	4,073 (34.5)	0.07
Liver Disease	649 (5.3)	1,381 (4.5)	3.56	605 (5.1)	605 (5.1)	0.00
Renal insufficiency	2,992 (24.4)	7,458 (24.5)	−0.08	2,897 (24.6)	2,877 (24.4)	0.39
COPD	1,358 (11.1)	3,294 (10.8)	0.91	1,287 (10.9)	1,295 (11.0)	−0.22
Heart failure	1,682 (13.7)	4,187 (13.7)	−0.00	1,624 (13.8)	1,593 (13.5)	0.77
Pneumonia	1,555 (12.7)	3,944 (12.9)	−0.72	1,498 (12.7)	1,471 (12.5)	0.69
**Cancer-Entity (%)**						
Gastrointestinal	3,641 (29.7)	10,135 (33.2)	−7.58	3,539 (30.0)	3,511 (29.8)	0.52
Respiratory	2,658 (21.7)	5,864 (19.2)	6.11	2,498 (21.2)	2,520 (21.4)	−0.46
Hematological	1,961 (16.0)	4,817 (15.8)	0.57	1,876 (15.9)	1,877 (15.9)	−0.02
Urogenital	1,992 (16.3)	4,686 (15.4)	2.45	1,928 (16.4)	1,907 (16.2)	0.48
Mamma	866 (7.1)	2,261 (7.4)	−1.33	846 (7.2)	832 (7.1)	0.46
Others	1,586 (12.9)	3,887 (12.7)	0.59	1,526 (12.9)	1,553 (13.2)	−0.68
**Therapies (%)**						
Chemotherapy	1,994 (16.3)	6,318 (20.7)	−11.46	1,962 (16.6)	1,918 (16.3)	1.01
Radiotherapy	1,185 (9.7)	4,028 (13.2)	−11.13	1,176 (10.0)	1,207 (10.2)	−0.87
Immunotherapy	803 (6.6)	2,262 (7.4)	−3.39	774 (6.6)	759 (6.4)	0.52
Palliative therapy	1,626 (13.3)	4,389 (14.4)	−3.25	1,566 (13.3)	1,618 (13.7)	−1.29
**General Health Status**						
Malnutrition severe (%)	1,612 (13.2)	11,123 (36.5)	−56.05	1,612 (13.7)	1,655 (14.0)	−1.06
**Total Amount of Hospitalizations (%)**						
1 time	809 (6.6)	2,231 (7.3)	6.71	792 (6.7%)	763 (6.5%)	−0.10
2–5 times	4,667 (38.1)	12,456 (40.8)		4,532 (38.4%)	4,583 (38.9%)	
>5 times	6,778 (55.3)	15,814 (51.8)		6,466 (54.8%)	6,444 (54.7%)	
Charlson Index, mean (*SD*)	6.8 (3.1)	6.8 (3.1)	0.33	6.8 (3.1)	6.8 (3.1)	0.35
**Hospital Frailty Score (%)**						
<5 points	8,210 (67.0)	19,667 (64.5)	−4.80	7,860 (66.7)	7,913 (67.1)	1.41
5–15 points	3,778 (30.8)	10,172 (33.3)		3,670 (31.1)	3,650 (31.0)	
>15 points	266 (2.2)	662 (2.2)		260 (2.2)	227 (1.9)	
LOS, median (IQR)	9 (5, 16)	13 (8, 21)	−27.47	10 (5, 17)	10 (6, 16)	0.24

*std, standardized difference; COPD, chronic obstructive lung disease; LOS, length of hospital stay*.

### Primary Endpoint

**Figure 2:**
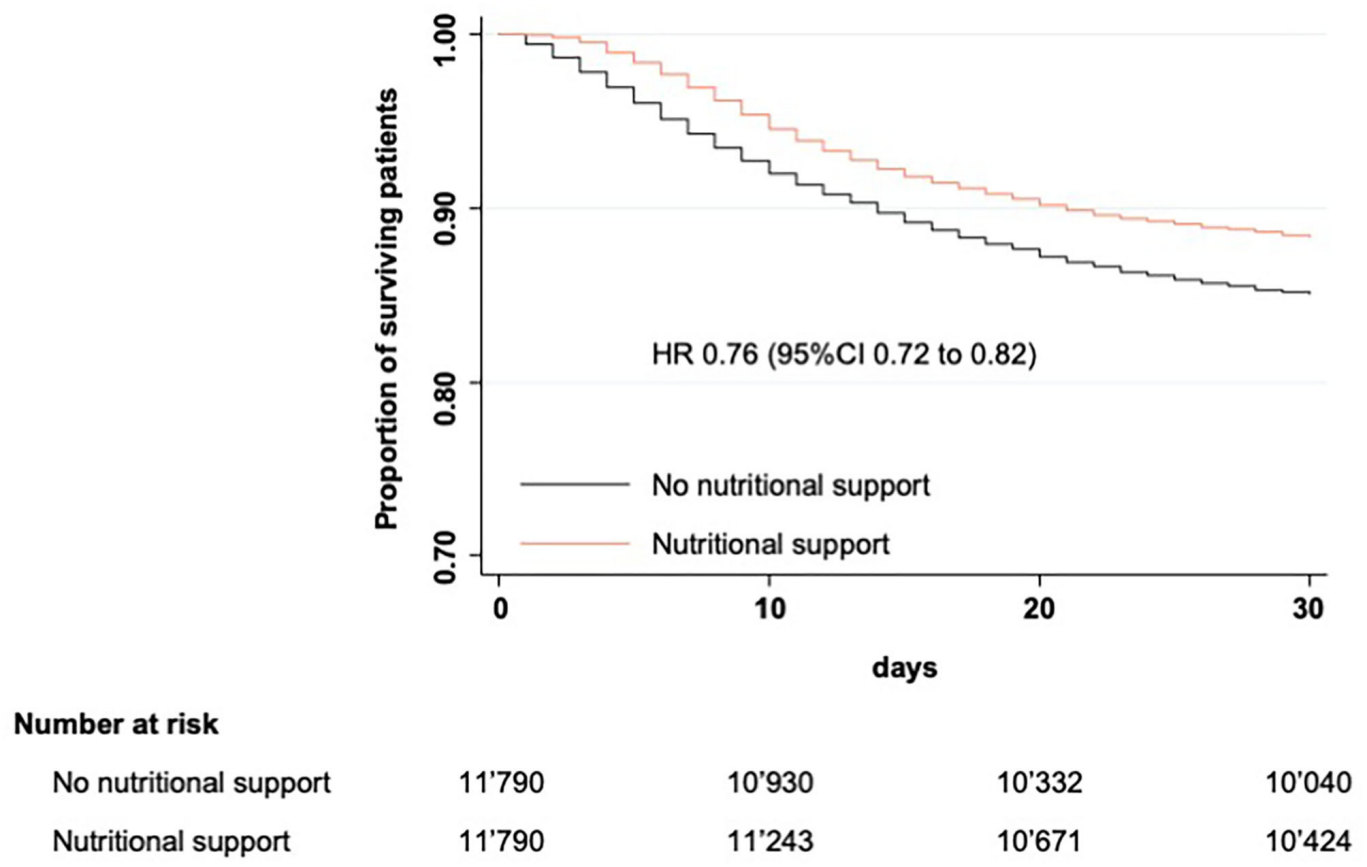
Kaplan-Meier estimates of cumulative incidence of all-cause in-hospital mortality.

In the unmatched study population, in-hospital mortality rate was 16.9% (2,065/12,254) in the control group and 14.4% (4,397/30,501) in the nutritional intervention group corresponding to an odds ratio (OR) of 0.83 with 95% confidence interval (CI) of 0.79–0.88 (Table 2). The association between nutritional support and reduced mortality rate remained significant in the adjusted and the fully adjusted model (OR 0.82, 95%CI 0.77–0.86 and OR 0.82, 95%CI 0.69–0.79, respectively). After propensity score matching, the overall in-hospital mortality rate was 14.9%. 1,548 (13.1%) patients on nutritional support died during hospitalization compared to 1,970 (16.7%) patients in the control group (OR 0.73, 95%CI 0.68–0.79). Survival benefit over 30 days of hospitalization is graphically illustrated in Figure 2.

**Table 2:** Association of nutritional support with clinical outcomes in the overall study population.

	**No nutritional support**	**Nutritional support**
**In-Hospital All-Cause Mortality**		
No of patients	12,254	30,501
No of events (%)	2,065 (16.9)	4,397 (14.4)
Unadjusted analysis (OR, 95%CI)	Ref	0.83 (0.79 to 0.88)
Adjusted analysis^a^ (OR, 95%CI)	Ref	0.82 (0.77 to 0.86)
Fully adjusted analysis^b^ (OR, 95%CI)	Ref	0.74 (0.69 to 0.79)
No of patients after PSM	11,790	11,790
No of events after PSM (%)	1,970 (16.7)	1,548 (13.1)
PSM analysis^c^ (OR, 95%CI)	Ref	0.73 (0.68 to 0.79)
**30-Days Readmission Rate**		
No of patients	10,189	26,104
No of events (%)	3,147 (25.7)	7,624 (25.0)
Unadjusted analysis (OR, 95%CI)	Ref	0.92 (0.88 to 0.97)
Adjusted analysis^a^ (OR, 95%CI)	Ref	0.92 (0.87 to 0.96)
Fully adjusted analysis^b^ (OR, 95%CI)	Ref	0.98 (0.93 to 1.03)
No of patients after PSM	9,820	10,242
No of events after PSM (%)	3,020 (30.8)	3,097 (30.2)
PSM analysis^c^ (OR, 95%CI)	Ref	0.99 (0.93 to 1.05)
**Discharge to Post-Acute Care Facility**		
No of patients	10,189	26,104
No of events (%)	3,422 (33.6)	8,587 (32.9)
Unadjusted analysis (OR, 95%CI)	Ref	0.97 (0.92 to 1.02)
Adjusted analysis^a^ (OR, 95%CI)	Ref	1.04 (0.99 to 1.09)
Fully adjusted analysis^b^ (OR, 95%CI)	Ref	0.90 (0.85 to 0.95)
No of patients after PSM	9,820	10,242
No of events after PSM (%)	3,285 (33.5)	3,164 (30.9)
PSM analysis^c^ (OR, 95%CI)	Ref	0.89 (0.84 to 0.94)

*OR, Odd Ratio; CI, Confidence Interval; PSM, propensity-score matching*.

a *adjusted for sociodemographic factors: age, gender, nationality, insurance status, mode of admission, place before admission, hospital size, hospital site, month and year of admission*.

b *adjusted for sociodemographic factors and medical factors: main diagnosis, comorbidities, severity of malnutrition, total amount of hospitalizations, palliative treatment, Chalson Comorbidity Index, Hospital Frailty Risk Score, and length of hospital stay*.

c *sociodemographic factors and medical factors used in the propensity score matching, analyses adjusted for hospital site*.

### Secondary Endpoints

In the unadjusted analysis, 30-days readmission rate was significantly lower in the nutritional intervention group compared with the control group (25.0% in patients with nutritional support vs. 25.7% in patients without nutritional support, OR 0.92, 95%CI 0.88–0.97). However, there was no significant association in the fully adjusted analysis nor in the propensity-score matched analysis, respectively.

Considering discharge to post-acute care facility, there was no difference in the unmatched population but after full adjustment and in the propensity-score matched cohort, patients receiving nutritional support during hospitalization were less likely to be admitted to a post-acute care facility as compared to control patients.

### Sensitivity and Subgroup Analyses

**Figure 3:**
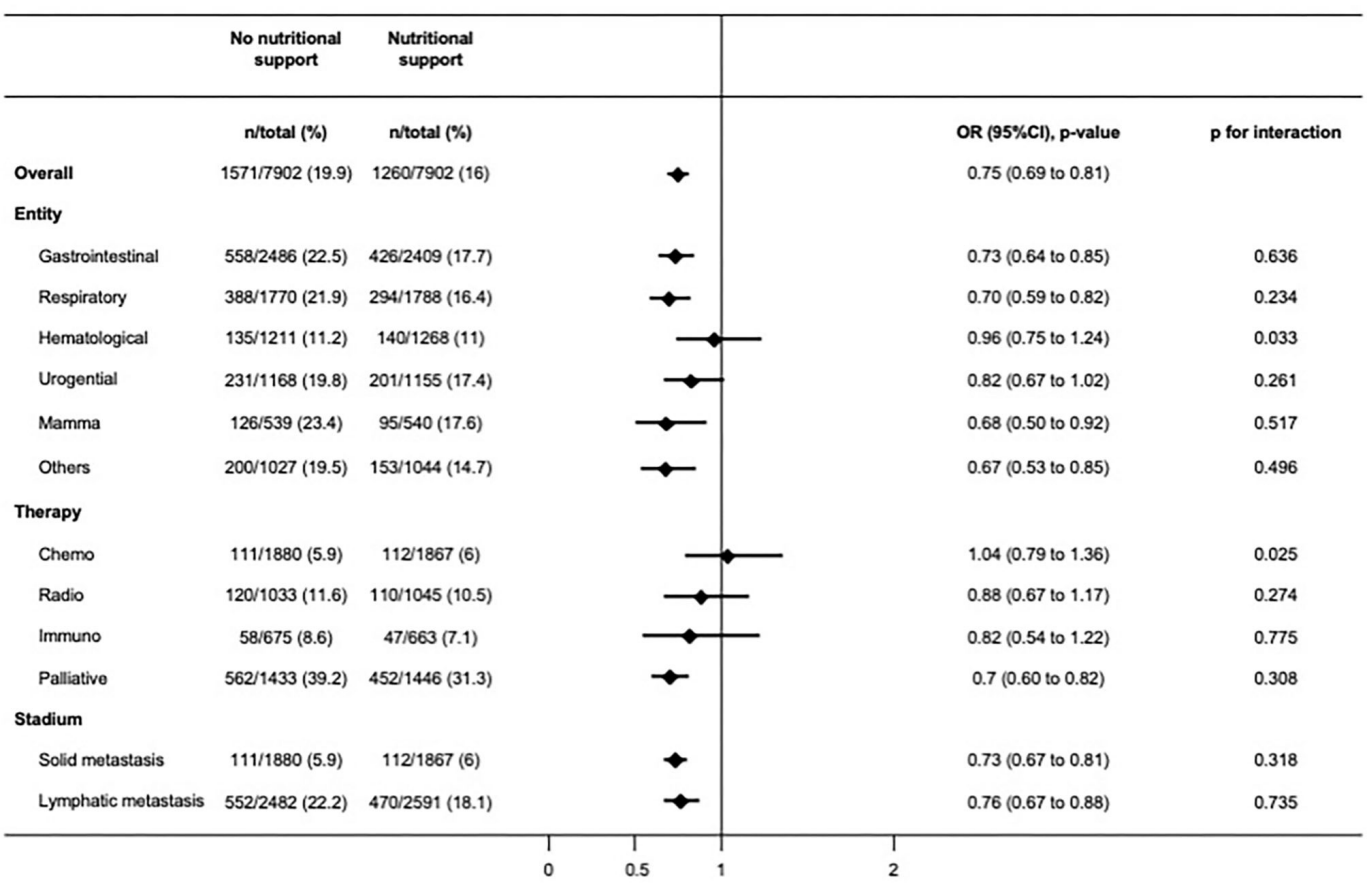
Subgroup analyses showing odds ratios for primary outcome after matching in patients admitted for an oncological diagnosis.

Among hospitalizations with cancer as main admission diagnosis, nutritional support was prescribed in 21,525 (72.1%) patients, while 8,336 (27.9%) patients did not receive nutritional support. Baseline characteristics before and after 1:1 propensity-score matching of this population are shown in Appendix Table 2. Results for mortality, readmission rate, and discharge to post-acute care facility remained robust in this cohort of patients with cancer as admission diagnosis compared to the overall population (Table 3). In a subgroup analysis of this population we stratified for cancer entity, cancer treatment, and cancer stadium (Figure 3). The findings for in-hospital mortality remained robust with two exceptions: patient groups with hematological cancer and on chemotherapy both did not show significant survival benefit (OR 0.96, 95%CI 0.75–1.24, *p* for interaction 0.033; OR 1.04, 95%CI 0.79–1.36, *p* for interaction 0.025, respectively).

**Table 3:** Association of nutritional support with clinical outcomes in patients admitted for an oncological diagnosis.

	**No nutritional support**	**Nutritional support**
**In-Hospital All-Cause Mortality**		
No of patients	8,336	21,525
No of events (%)	1,677	3,706
Unadjusted analysis (OR, 95%CI)	20.1	17.2
Adjusted analysis^a^ (OR, 95%CI)	Ref	0.83 (0.77 to 0.88)
Fully adjusted analysis^b^ (OR, 95%CI)	Ref	0.81 (0.75 to 0.86)
No of patients after PSM	7,902	7,902
No of events after PSM (%)	1,571 (19.9)	1,260 (16.0)
PSM analysis^c^ (OR, 95%CI)	Ref	0.75 (0.69 to 0.81)
**30-Days Readmission Rate**		
No of patients	6,659	17,819
No of events (%)	2,347	5,796
Unadjusted analysis (OR, 95%CI)	35.3	32.5
Adjusted analysis^a^ (OR, 95%CI)	Ref	0.89 (0.83 to 0.94)
Fully adjusted analysis^b^ (OR, 95%CI)	Ref	0.89 (0.83 to 0.94)
No of patients after PSM	6,331	6,642
No of events after PSM (%)	2,218 (35.0)	2,288 (34.5)
PSM analysis^c^ (OR, 95%CI)	Ref	0.99 (0.92 to 1.06)
**Discharge to Post-Acute Care Facility**		
No of patients	6,659	17,819
No of events (%)	2,126	5,639
Unadjusted analysis (OR, 95%CI)	31.93	31.65
Adjusted analysis^a^ (OR, 95%CI)	Ref	0.99 (0.93 to 1.05)
Fully adjusted analysis^b^ (OR, 95%CI)	Ref	1.05 (0.98 to 1.12)
No of patients after PSM	6,331	6,642
No of events after PSM (%)	2,002 (31.6)	1,917 (28.9)
PSM analysis^c^ (OR, 95%CI)	Ref	0.87 (0.81 to 0.94)

*OR, Odd Ratio; CI, Confidence Interval; PSM, propensity-score matching*.

a *adjusted for sociodemographic factors: age, gender, nationality, insurance status, mode of admission, place before admission, hospital size, hospital site, month and year of admission*.

b *adjusted for sociodemographic factors and medical factors: main diagnosis, comorbidities, severity of malnutrition, total amount of hospitalizations, palliative treatment, Chalson Comorbidity Index, Hospital Frailty Risk Score, and length of hospital stay*.

c *sociodemographic factors and medical factors used in the propensity score matching, analyses adjusted for hospital site*.

## Discussion

This large population-based cohort study revealed two key findings. First, inhospital mortality rate was lower among malnourished cancer patients who received nutritional support as compared to those not receiving additional nutritional support. Results were consistent in patients mainly admitted for cancer *per se*. Second, the findings of this study remained robust among the different cancer entities and treatment options except for patients suffering from a hematological cancer or undergoing chemotherapy.

To the best of our knowledge, there is only few data investigating the association of nutritional intervention and mortality in a heterogenous cancer patient population specifically. Previous studies have focused rather on general medical patients including cancer patients, but evidence still remains scarce. For example, the EFFORT trial, which reported an overall 35% relative risk reduction for 30-days mortality in patients receiving nutritional support vs. standard care did not find any significant risk reduction in the subgroup of cancer patients (4). In contrast to our study, most of the studies in cancer patients are from the outpatient setting. For example a smaller study—specifically investigating colorectal cancer patients—reported that patients with individualized nutritional counseling or with nutritional supplement therapy showed a long-term survival benefit as compared with the control group receiving usual care (13). Considering parenteral nutrition, there are few randomized controlled trials but results are heterogenous. No beneficial or even detrimental effects (more adverse events) were found in a study with advanced colorectal cancer patients (16), whereas in another study with a similar population there was an effect on fat-free mass and quality of life (17). Nonetheless, no studies reported from real-world cancer inpatients as treated in clinical routine, limiting the comparability of the studies.

We found no significant reduction in readmission rates associated with nutritional support. Similarly, Britton et al. (18) did not find any significant benefit regarding readmission rates in a population of head and neck cancer patients comparing a psychological intervention to improve nutritional intake to standard dietician treatment. In contrast, a retrospective study also investigating head and neck cancer patients showed a significantly lower readmission rate in patients undergoing prophylactic gastrostomy to improve nutritional status (18). However, their intervention was more invasive and the retrospective design might introduce confounding by indication. Regarding discharge to post-acute care facilities, we found the patients on nutritional support being less frequently discharged to a post-acute care facility. Taking into account the limited places in those facilities, the increasing health care costs and patients preferences, this finding may be of particular interest for physicians, policy makers and patients. So far, no further studies have investigated this outcome.

Assessing the association of nutritional support among patients primarily hospitalized for an oncological reason, the in-hospital mortality benefit of nutritional support was present in most of the different cancer entities and treatment options, with, however, two exceptions, hematological malignancies, and chemotherapy. Most of the previous trials investigating hematological malignancies were performed in patients on chemotherapy. A meta-analysis published in 2018 included 11 trial of cancer patients with chemo(radio)therapy and assessed the effect of nutritional interventions on body weight (12). They found a significant effect of nutritional interventions including dietary advice, ONS and ONS enriched with protein and n-3 polyunsaturated fatty acids (PUFA) on body weight (1.31 kg, 95% CI 0.24–2.38, *P* 0.02, heterogeneity *Q* = 21.1, *P* = 0.007), mainly driven by high-protein n-3 PUFA-enriched ONS. But they did not show any effect on survival. Limiting factors of this meta-analysis were high heterogeneity in treatment effects, low to moderate quality of the included studies, and inclusion of different settings as well as different interventions (19). Another nutritional treatment option for patients with chemotherapy is glutamine. In the review of Kuhn et al. they reported that eight of 24 studies using oral, and six of 12 studies using parenteral glutamine found a clinical benefit (i.e., decreased risks of high-dose chemotherapy and radiation). But, according to the current ESPEN guidelines (10) the role of glutamine in chemotherapy patients remains controversial.

In summary, evidence in patients with hematological cancer entities and on chemotherapy is still poor and leads to the suggestion that provision of the right nutrients with the right application form may be crucial. This underlines the importance of future well-designed clinical trials for nutritional interventions in (hematological) cancer patients undergoing chemotherapy.

The strengths and limitations of this study has to be interpreted in the context of the study design. The main strength of this study is that it includes a nation-wide sample of all hospitalized cancer patients in Switzerland between 2013 and 2018. Because of the heterogeneity of the population and in regard to robust results in the sensitivity and subgroup analyses, our findings reveal high external validity. Additionally, we were able to address multiple possible confounding variables by including them into our adjusted or propensity-score matched analyses. The definition of nutritional support in this study is highly specific, but due to the possibility of missed coding for nutritional support, there could be some patients in the control group also receiving nutritional support, mirroring decreased sensitivity. We thus might argue that the observed findings could be even underestimated.

The main limitations of this study involve, first, a certain risk of misclassification and underreporting of tumor entities, therapy forms, and malnutrition *per se* as we used ICD-10 codes. In our cohort of malnourished medical inpatients, the prevalence rate of malignant diseases was 30% which was similar to the results of the large multi-center EFFORT trial (4). Regarding the prevalence of malnutrition defined by ICD-10 code in the overall population of Swiss medical inpatients (6%), it was remarkably lower compared to trials using clinical screening tools [i.e., 28% in the study of Felder et al. (20)]. As shown in a former Swiss study investigating the diagnostic accuracy of malnutrition codes, sensitivity was low (30%) but specificity was high (93.4%) meaning that mostly all patients with a coding for malnutrition really were malnourished but a relevant proportion of patients with malnutrition might not be included in our study (21). Similar, we were not able to assess activity and severity of the malignant disease nor the treatment strategy in detail. But malignancies which did not affect the actual state of health or which were considered as cured had to be coded with a different ICD-10-GM code, which we did not include in our analysis. Also, the high average inhospital mortality rate of 15.6% could be an indicator that our population in fact included patients with more severe neoplastic diseases. Additionally, our sensitivity analysis only including patients with an oncological admission diagnosis showed robust results. Second, type of nutritional intervention was not defined in detail by the CHOP codes and the attribution of the nutritional intervention was in discretion of the treating physician. Third, unmeasured or unmeasurable confounding cannot be excluded, and whether more palliative patients did not qualify for an additional therapy anymore, should be critically discussed. However, we included several surrogate variables concerning general health status (Hospital Frailty Index, presence of a code for palliative treatment, burden of comorbidities) into our statistical models to better account for potential residual confounding.

## Conclusion

In this large nationwide cohort study of malnourished cancer patients, we found nutritional support as prescribed in clinical practice to be associated with a decrease in in-hospital mortality. These findings support the current guidelines, which recommend a standardized screening and multidisciplinary management of malnutrition in cancer patients. By broadening the use of a validated screening tool [i.e., NRS 2002 (22)], the awareness of physicians and nurses will further increase and consequently more patients at risk for malnutrition could benefit from an early treatment performed by trained dieticians. Further high-quality studies are needed to investigate optimal disease and treatment-specific nutritional support to individualize the treatment of malnourished cancer patients.

## Data Availability Statement

The datasets generated for this study are available on request to the corresponding author.

## Ethics Statement

The studies involving human participants were reviewed and approved by Institutional review board of Northwestern Switzerland (AG/SO 2009/074 and EKNZ BASEC PB_2017-00449). Written informed consent for participation was not required for this study in accordance with the national legislation and the institutional requirements.

## Author Contributions

NK-B and AK designed the study, wrote the manuscript, and analyzed the data. AK had full access to all the data. All authors were responsible for the decision to submit the manuscript, provided substantial comments on drafts, and approved the final report.

## Conflict of Interest

The authors declare that the research was conducted in the absence of any commercial or financial relationships that could be construed as a potential conflict of interest.

## Footnotes

^1^*Medizinische Statistik der Krankenhäuser*. (2020). Available online at: https://www.bfs.admin.ch/bfs/de/home/statistiken/gesundheit/erhebungen/ms.assetdetail.7369.html (accessed July 11, 2020).

^2^*Online Definitionshandbuch*. (2020). Available online at: https://manual50.swissdrg.org (accessed July 11, 2020).
