# Supplementary material for: Association of Nutritional Support With Clinical Outcomes in Malnourished Cancer Patients: A Population-Based Matched Cohort Study
Source: Front Nutr. 2021 Mar 10;7:603370. doi: 10.3389/fnut.2020.603370 (PMC7987808; doi:10.3389/fnut.2020.603370)
Supplement: Supplementary file 1 [file Table_1.docx]

**Appendix**

**Appendix Table 1: Detailed information about ICD-10-GM codes of malnutrition (implemented in March 2013)**

| **ICD-10-GM Codes** | **Definition** | **Criteria** | **Condition for coding** |
| --- | --- | --- | --- |
|  |  |  |  |
| **E43** | **Unspecified severe protein-energy malnutrition** | NRS 2002^1)^ ≥ 5  and  one of the following criteria   - BMI <18.5kg/m^2^ - Unintended weight loss of >5% in 1 month and reduced general health - Reduced food intake (0-25% of the daily needs) | Combination with a CHOP code for a nutritional intervention |
| **E44** | **Protein-energy malnutrition of moderate and mild degree** | E44.0 moderate:  NRS 2002 ≥ 4  and  one of the following criteria   - BMI 18.5-20.5kg/m^2^ - Unintended weight loss of >5% in 2 month and reduced general health - Reduced food intake (25-50% of the daily needs)   E44.1 mild:  NRS 2002 ≥ 3  and  one of the following criteria   - Unintended weight loss of >5% in 3 month and reduced general health - Reduced food intake (50-75% of the daily needs) | Combination with a CHOP code for a nutritional intervention |
| **E46** | **Unspecified protein-energy malnutrition** | Patients who do not fulfill the criteria above or who did not have a CHOP-code for nutritional support | No conditions |

^1)^ NRS 2002: nutritional risk screening[1]

**Appendix Table 2: Baseline characteristics of patients with oncological admission diagnosis before and after 1:1 propensity-score matching stratified by nutritional support**

| **Population with oncological admission diagnosis** | | | | | | | |
| --- | --- | --- | --- | --- | --- | --- | --- |
|  | Before matching | |  |  | After matching | |  |
|  | No nutritional support | Nutritional support | Std (%) |  | No nutritional support | Nutritional support | Std (%) |
| n | 8’336 | 21’525 |  |  | 7’902 | 7’902 |  |
| **Sociodemographics** |  |  |  |  |  |  |  |
| Age, mean (SD) | 69.1 (13.1) | 68.4 (13.0) | 4.85 |  | 68.9 (13.2) | 69.0 (12.6) | -0.31 |
| Female (%) | 3’733 (44.8) | 9’743 (45.3) | -0.97 |  | 3’527 (44.6) | 3’555 (45.0) | -0.71 |
| Swiss resident (%) | 6’968 (83.6) | 18’253 (84.8) | 3.32 |  | 6’613 (83.7) | 6’583 (83.3) | -1.02 |
| Public insurance (%) | 6’330 (75.9) | 16’451 (76.4) | 1.15 |  | 6’014 (76.1) | 5’951 (75.3) | -1.86 |
| Emergency admission (%) | 4’675 (56.1) | 13’332 (61.9) | -11.93 |  | 4’540 (57.5) | 4’527 (57.3) | 0.33 |
| Admission from home (%) | 6’922 (83.0) | 18’886 (87.7) | -13.34 |  | 6’684 (84.6) | 6’687 (84.6) | -0.11 |
| Tertiary hospital (%) | 6’404 (76.8) | 18’477 (85.8) | -23.29 |  | 6’256 (79.2) | 6’203 (78.5) | 1.64 |
| **Comorbidities (%)** |  |  |  |  |  |  |  |
| Diabetes | 1’227 (14.7) | 3’220 (15.0) | -0.68 |  | 1’186 (15.0) | 1’184 (15.0) | 0.07 |
| Coronary heart disease | 770 (9.2) | 2’221 (10.3) | -3.64 |  | 749 (9.5) | 722 (9.1) | 1.18 |
| Hypertension | 2’634 (31.6) | 7’352 (34.2) | -5.45 |  | 2’561 (32.4) | 2’551 (32.3) | 0.27 |
| Liver Disease | 397 (4.8) | 915 (4.3) | 2.47 |  | 370 (4.7) | 374 (4.7) | -0.24 |
| Renal insufficiency | 1’682 (20.2) | 4’644 (21.6) | -3.44 |  | 1’623 (20.5) | 1’658 (21.0) | -1.09 |
| COPD | 810 (9.7) | 2’047 (9.5) | 0.70 |  | 748 (9.5) | 742 (9.4) | 0.26 |
| Heart failure | 897 (10.8) | 2’421 (11.2) | -1.56 |  | 867 (11.0) | 849 (10.7) | 0.73 |
| Pneumonia | 776 (9.3) | 2’220 (10.3) | -3.38 |  | 743 (9.4) | 714 (9.0) | 1.27 |
| **Cancer-entity (%)** |  |  |  |  |  |  |  |
| Gastrointestinal | 2’574 (30.9) | 7’464 (34.7) | -8.10 |  | 2’486 (31.5) | 2’409 (30.5) | 2.11 |
| Respiratory | 1’918 (23.0) | 4’352 (20.2) | 6.78 |  | 1’770 (22.4) | 1’788 (22.6) | -0.55 |
| Hematological | 1’292 (15.5) | 3’341 (15.5) | -0.06 |  | 1’211 (15.3) | 1’268 (16.0) | -1.98 |
| Urogenital | 1’229 (14.7) | 3’067 (14.2) | 1.41 |  | 1’168 (14.8) | 1’155 (14.6) | 0.47 |
| Mamma | 571 (6.8) | 1’445 (6.7) | 0.54 |  | 539 (6.8) | 540 (6.8) | -0.05 |
| Others | 1’074 (12.9) | 2’701 (12.5) | 1.01 |  | 1’027 (13.0) | 1’044 (13.2) | -0.64 |
| **Therapies (%)** |  |  |  |  |  |  |  |
| Chemotherapy | 1’929 (23.1) | 6’072 (28.2) | -11.62 |  | 1’880 (23.8) | 1’867 (23.6) | 0.39 |
| Radiotherapy | 1’043 (12.5) | 3’664 (17.0) | -12.74 |  | 1’033 (13.1) | 1’045 (13.2) | -0.45 |
| Immunotherapy | 725 (8.7) | 1’947 (9.0) | -1.22 |  | 675 (8.5) | 663 (8.4) | 0.55 |
| Palliative therapy | 1’524 (18.3) | 4’066 (18.9) | -1.56 |  | 1’433 (18.1) | 1’446 (18.3) | -0.43 |
| **General health status** |  |  |  |  |  |  |  |
| Malnutrition severe (%) | 1’134 (13.6) | 7’832 (36.4) | -54.54 |  | 1’134 (14.4) | 1’201 (15.2) | -2.39 |
| Total amount of hospitalizations (%) |  |  |  |  |  |  |  |
| 1 time | 618 (7.4) | 1’789 (8.3) | 8.10 |  | 602 (7.6) | 573 (7.3) | 0.66 |
| 2-5 times | 3’297 (39.6) | 9’232 (42.9) |  |  | 3’149 (39.9) | 3’240 (41.0) |  |
| >5 times | 4’421 (53.0) | 10’504 (48.8) |  |  | 4’151 (52.5) | 4’089 (51.7) |  |
| Charlson Index (SD) | 7.1 (3.0) | 7.1 (3.0) | 0.22 |  | 7.1 (3.0) | 7.0 (3.0) | 1.31 |
| Hospital Frailty score (%) |  |  |  |  |  |  |  |
| <5 points | 6’052 (72.6) | 14’875 (69.1) | -7.44 |  | 5’695 (72.1) | 5’757 (72.9) | 1.96 |
| 5-15 points | 2’176 (26.1) | 6’341 (29.5) |  |  | 2’105 (26.6) | 2’055 (26.0) |  |
| >15 points | 108 (1.3) | 309 (1.4) |  |  | 102 (1.3) | 90 (1.1) |  |
| LOS (median, IQR) | 10 (5, 18) | 14 (8, 22) | -29.71 |  | 10 (5, 18) | 10 (6, 17) | 0.49 |

Abbreviations: std: standardized difference, COPD: chronic obstructive lung disease, LOS: length of hospital stay

[1] Kondrup J, Rasmussen HH, Hamberg O, Stanga Z. Nutritional risk screening (NRS 2002): a new method based on an analysis of controlled clinical trials. Clinical nutrition (Edinburgh, Scotland). 2003;22:321-36.
